# Scale validation for the identification of falsified hand sanitizer: public and regulatory authorities perspectives from United Arab Emirates

**DOI:** 10.1186/s12889-020-09707-0

**Published:** 2020-10-22

**Authors:** Ammar Abdulrahman Jairoun, Sabaa Saleh Al-Hemyari, Moyad Shahwan, Faris El-Dahiyat, Shazia Jamshed

**Affiliations:** 1 Health and Safety Department, Dubai Municipality, Dubai, UAE; 2 Pharmacy Department, Ministry of Health and Prevention, Dubai, UAE; 3 College of Pharmacy and Health Sciences, Ajman University, Ajman, UAE; 4 College of Pharmacy, Al Ain University, Al Ain, UAE; 5 Clinical Pharmacy and Practice, Faculty of Pharmacy, Besut Campus, uniSZA, Terengganu, Malaysia; 6 Pharmacy Practice, Kulliyyah of Pharmacy, International Islamic University Malaysia, Kuantan, Pahang Malaysia

**Keywords:** Falsified hand sanitizer, COVID-19, Validation studies, Reliability analysis, Counterfeit, Regulation and compliance behaviours

## Abstract

**Background:**

Since the time of declaration of global pandemic of COVID-19 by World Health Organization (WHO), falsified hand sanitizers surfaced regularly in markets, posing possible harm to public due to unlisted inclusion of methanol. The current research is an attempt to develop and validate a tool to document falsified hand sanitizer in the UAE community.

**Method:**

A descriptive cross-sectional community-based study was conducted among 1280 randomly selected participants. Respondents were sent a web-based electronic link to the survey via email. Content validity, factor analyses and known group validity were used to develop and validate a new scale to identify falsified hand sanitizer. Test-retest reliability, internal consistency, item internal consistency (IIC), and intraclass correlation coefficients (ICCs) were used to assess the reliability of the scale. SPSS version 24 was used to conduct data analysis.

**Results:**

A total of 1280 participants were enrolled in the study. The content validity index (CVI) was 0.83 with the final scale of 12 items. The Kaiser-Meyer-Olkin (KMO) value was 0.788, with the Bartlett test of sphericity achieving statistical significance (*p* < 0.001). Our factor analysis revealed a 3-component model. The 3-factor solution was confirmed by PCFA analysis and had associations with good fit values. The PCFA for NFI was 0.970, CFI 0.978, and TLI 0.967. All values were in excess of 0.95, with RMSEA values below 0.06 at 0.03; all of these values indicated a good model fit. The Cronbach’s alpha was good overall (0.867). All factors had a Cronbach’s alpha value in excess of 0.70. The instrument demonstrated that every item met the IIC correlation standard ≥0.40. The scale displayed good overall ICC statistics of 0.867 (95% CI 0.856–0.877) with statistical significance (*p* < 0.001). The scale’s test-retest reliability was assessed through correlation of the falsified hand sanitizer identification score of respondents at the two time points. The test-retest correlation coefficient was 0.770 (*p* value < 0.01). Participants with post-graduate education were more likely to identify the falsified hand sanitizer compared to those with high school education. (*p* < 0.001).

**Conclusions:**

This study developed and validated a new scale for the measurement of falsified hand sanitizer. This is expected to improve and promote collaboration between the health regulators and the public and hereby encourage customer satisfaction and participation.

## Background

The main ingredients of alcohol-based hand sanitizers are ethanol, isopropyl alcohol and N-propyl alcohol (or some combination of them), glycerin, perfume, and aqua [1]. These substances may be toxic to humans if used inappropriately. One example is that following either deliberate or accidental ingestion, isopropyl alcohol suppresses the respiratory and central nervous systems more severely than ethanol [2]. Since the COVID-19 pandemic began, the amount of hand sanitizer generally available has significantly decreased because the raw materials to manufacture these products are in short supply. The demand for such products from healthcare workers and the general public far outstripped the amount manufacturers could produce. As a result, many unregulated hand sanitizers have entered the market. This increase is partly attributable to poor manufacturing processes in the factories that make these products, which meant that they could not cope with the enormous increase in demand. Numerous non-specialist manufacturers began reorienting their activities towards the production of hand sanitizer.

There are a number of different categories of unregulated/falsified hand sanitizers. One type is characterized by the addition of methanol to the product even though the label states that it only contains ethanol. Numerous global reports have found that alcohol-based hand sanitizers sometimes contain undeclared methanol [3–5].

Methanol is toxic when inhaled, taken orally, or applied to the skin [6] and should never be employed in any hand hygiene product. Following occupational or non-occupational exposure, severe systemic toxicity and even mortality may occur [7–11]. Occasional instances of humans being poisoned [3] indicate that this type of hazardous product sometimes appears in the marketplace. Methanol in hand sanitizer is a significant danger to public health, especially as this type of product is frequently employed in healthcare and community care environments.

Another type of unregulated/falsified hand sterilizer is manufactured with an alcohol level below 60%, meaning that it is not effective for eliminating germs. In the United Arab Emirates, numerous inspections and reports have concluded that a significant quantity of the hand sanitizer sold in the Emirate is counterfeit.

Recently, a safety survey was undertaken in Dubai, UAE, in which 6/102 alcohol-based hand sanitizers tested were shown to have undeclared or unlisted methanol among the ingredients; others were shown to have alcohol content well below 60% even though their labels claimed alcohol content of 70% [12]. A similar inspection in Ajman, UAE, resulted in two factories being shut down after a significant quantity of fake medical sterilizer was seized. It was found that when the stickers were removed from the products, the actual product was a perfume spray for the body and not a medically sterile product as claimed [13].

The CDC has highlighted the importance of hand hygiene with the global emergence of COVID-19 and WHO already validated that hand sanitizer formulations incapacitate the virus causing COVID-19. WHO also advocated alcohol-based sanitizer formulations to thwart the proliferation of pathogens and research from Germany and Switzerland already reported sanitizers’ effectiveness against SARS-CoV-2. Similarly, a retrospective study in a radiology unit in Italy demonstrated applying adequate preventive actions including hand hygiene measures can effectively diminish the proliferation of of SARS-CoV-2 transmission among the interventional radiology staff [14]. Okunlola et al also emphasized the use of hand sanitizers in controlling virus transmission [15].

To be precise, the point of concern is the presence of methanol (wood alcohol) which is traced as unlisted ingredient in some sanitizers and therefore, can pose harm to skin when rubbed or life-threatening issues if ingested. In the light of the above-mentioned facts and research it is imperative to conduct the current research for establishing a tool that will help the consumers in determining the safe and effective hand sanitizer.

## Methods

### Study design/setting

This study was a descriptive cross-sectional community-based study conducted among Ajman University (AU) students and staff to develop and validate a novel self-reporting scale in the English language to measure the identification rate of falsified hand sanitizer among the public in the UAE. Respondents were sent a survey link by email, and data collection occurred between March 3, 2020 and March 25, 2020.

### Study participants (inclusion/exclusion criteria)

The target population for this research was any resident of the UAE, national or non-national, aged 18 or over, who was willing to participate. Participants aged less than 18 years and those who did not want to participate were excluded.

### Pilot testing

The pilot study began at Ajman University on October 20, 2019. As of November 1, 2019, 350 respondents had undertaken satisfactory completion of the questionnaire with no apparent difficulty. The outcomes of the pilot study were employed to calculate the sample size needed for the main research and to check the reliability of the test.

### Sample size/sampling technique

To calculate a sample size for this survey, a pilot study was used. The questionnaire was sent to 400 students and staff at Ajman University, from which 350 respondents were achieved, yielding a response rate of 87.5%. The sample size calculation was based on the question, ‘Do you know how to identify falsified hand sanitizer?’ According to the pilot study, the proportion of people who answered yes to this question was approximately 55%. The alpha level was set at 5%, giving a 95% confidence interval. Precision (D) for the 95% confidence interval was fixed at 5% so that the 95% CI would have a maximum width of 10%. On the basis of these assumptions, a sample size n of 1270 was required, assuming that nonresponse rates would be approximately 70%. Ajman University’s Admissions and Registration Department supplied us with an Excel spreadsheet containing the names of students and staff and their colleges, study year, and email addresses. Basic random-sample selection was used to choose the sample, with ID numbers employed for random selection, stratified by department and college. A total of 1280 participants were chosen for the final sample.

### Administration of questionnaires

The survey instrument was designed as a self-administered survey to be used by preselected respondents chosen at random from the spreadsheet provided by the Admissions and Registration Department. Participants were sent a web-based electronic link to the survey via email. The first page of the survey comprised an explanation of the type of and reason for the study. When participants moved to the next page, this was registered as their consent to participate. Reminder emails were sent two times in a month from the inception of the survey. On the completion of survey the respondents were acknowledged with a ‘thank you’ message and were not given any incentives.

### Research instrument conceptualization/development

#### Face/content validity

The questionnaire’s first draft was subjected to face and content validity testing. An expert panel was convened comprising an industrial pharmacist, an academic, two regulatory pharmacists, and two community pharmacists. The panel assessed the content validity of the scale. Additionally, public consultations formed part of the process. The content validity ratio and content validity index (CVI/CVR) were calculated by requesting that the experts classify every item in the instrument as essential or nonessential. Good content validity is demonstrated when the CVR reaches 0.78 or above. Any individual item that does not meet this level would generally be removed from the final draft. The CVI was then calculated by taking the mean of all CVR values for all items that were kept in the final draft, i.e., those with a CVR above 0.78 [16, 17].

#### Construct validity

Exploratory factor analysis (EFA) was used to test construct validity. Factor analysis was undertaken using principal component analysis (PCA); then, varimax rotation with Kaiser-Mayer-Olkin (KMO) and Bartlett’s sphericity test were conducted to assess factor numbers. The construct validity criteria were satisfied when there were eigenvalues of 1 and item loadings of 0.40 or above with no cross-loading [18]. Confirmation of the model was then performed using partial confirmatory factor analysis (PCFA) employing maximum likelihood analysis using oblimin rotation. Calculations were undertaken of the incremental fit indices of the Tucker-Lewis index (TLI), normed fit index (NFI), and comparative fit index (CFI). In addition, we reported the absolute fit index using root mean square error of approximation (RMSEA) [19, 20].

### Internal consistency/reliability analysis

Intraclass correlation coefficients (ICCs) and Cronbach alpha was computed to ascertain test-retest reliability and internal consistency. A value of 0.7 or above is acceptable for Cronbach alpha [21]. For ICCs which is a ratio also, is considered highly reliable when the value is near to 1. In the current research Rosner’s criteria is employed to interpret ICCs, which reflects ICC < 0.40 corresponds to poor agreement, 0.40 ≤ ICC < 0.75 corresponds to fair/good agreement and ICC ≥ 0.75 relates to excellent agreement [22]. For the estimation of item internal consistency (IIC) Pearson correlation was employed. IIC involves the relationship every item has with its hypothesized domain or factor. The IIC demands that the adjusted scale score should correlate with the item r ≥ 0.4 [23]. Test-retest reliability across two points in time was assessed following a six-week gap using Pearson’s correlation coefficient (ρ). A (ρ) value above 0.75 and a *p*-value < 0.05 are regarded as a correlation with strong significance [19, 20].

### Known group validation

We hypothesized that respondents with higher levels of education would have a better ability to identify falsified hand sanitizer than those with lower levels of education. A one-way ANOVA test was used.

### Statistical analysis

SPSS version 24 was used to conduct data analysis. Frequencies and percentages were employed to summarize the demographic/baseline characteristics from the study sample. One-way ANOVA was used to calculate the correlation between demographics and the ability to identify falsified hand sanitizer. A *p*-value below 0.05 was regarded as statistically significant.

## Results

### Demographic/baseline characteristics of study participants

A total of 1280 participants were enrolled in the study and completed the entire questionnaire after providing their consent, out of which 350 participants returned completed questionnaires twice. Among these participants, 55% (*n* = 704) were male and 45% (*n* = 576) were female. Regarding age, 11.3% were aged 18–24, 21.9% were aged 25–34, 20.6% were aged 35–44, 31.9% were aged 45–54 and 14.4% were aged ≥55. The Middle East (*n* = 752, 58.8%) constituted the origin of the largest ethnic group in the study, followed by Africa (*n* = 304, 23.8%), UAE (*n* = 96, 7.5%), Asia (*n* = 88, 6.9%) and America (*n* = 40, 3.1%). Most of the participants in the study were employed (*n* = 960, or 75%). The educational qualifications of the participants varied. Approximately 31.3% (*n* = 400) were high school education holders, 66.3% (*n* = 848) held bachelor’s certificates, and 2.5% (*n* = 32) were postgraduate (Table 1).

**Table 1:** Number and percentage of questions on baseline characteristics (*n* = 1280)

Variable	Groups	Frequency	Percentage
Sex	Male	704	55%
	Female	576	45%
Age	18–24	144	11.3%
	25–34	280	21.9%
	35–44	264	20.6%
	45–54	408	31.9%
	≥ 55	184	14.4%
Nationality	UAE	96	7.5%
	Asia	88	6.9%
	Africa	304	23.8%
	America	40	3.1%
	Middle East	752	58.8%
Education	High school	400	31.3%
	Bachelor’s degree	848	66.3%
	Postgraduate	32	2.5%
Employment status	Employed	960	75%
	Unemployed	320	25%

### Validation analysis

#### Face/content validity

There were 15 items in the first draft of the scale, which was then scrutinized by an expert panel. Three of the items underwent modification. A content validity ratio (CVR) of 0.78 was needed for an item to be retained. Two items were removed on the basis of low CVR. Once these two items were removed, the content validity index (CVI) was 0.83, with the final scale of 12 items.

#### Construct validity (factor analysis)

**Fig. 1:**
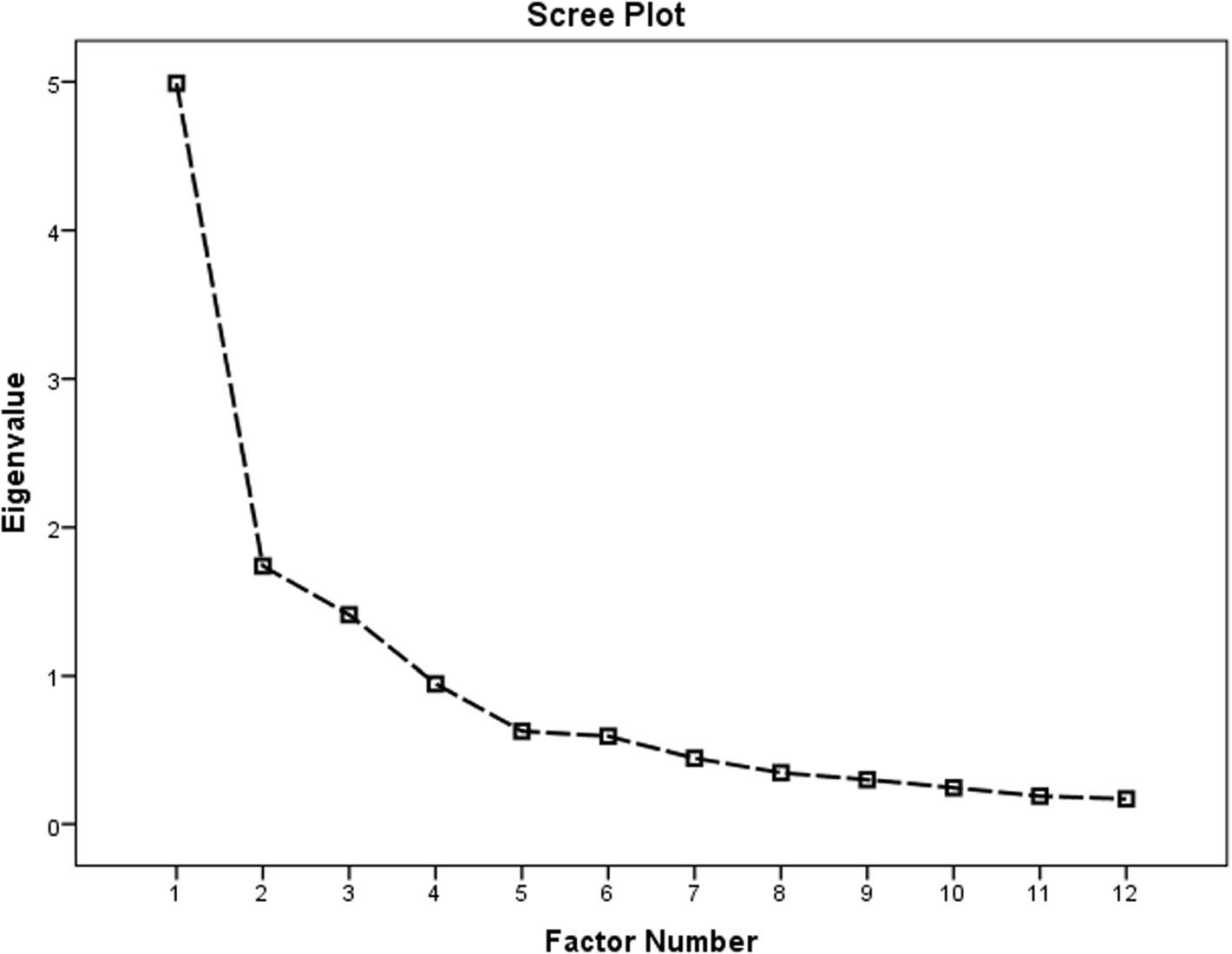
Scree plot and component factors resulting from PCA

The scale’s factor structure underwent analysis via EFA employing PCA and varimax rotation. The Kaiser-Mayer-Olkin (KMO) measure for sampling adequacy was 0.812, and Bartlett’s test of sphericity had a *p*-value < 0.001. A total of 67.8% of the variance was accounted for by eigenvalues > 1 obtained using a 3-factor solution. Factor 1 constituted 41.6% of the variance, factor 2 14.5%, and factor 3 11.8%. Items were regarded as a single factor if they had factor loading > 0.4 on one component and non-salient loading < 0.4 on another. This showed a clear factor structure (Table 2/Fig. 1).

**Table 2:** Principal component analysis of Safety, Identity and Efficacy Measures

	Item content	CVR	Component			Communalities
			1	2	3	
Factor 1: Safety Measure	How to use/usage directions	0.83	**0.875**	0.064	0.147	0.791
	Warnings/cautions/hazard pictogram	0.84	**0.849**	0.205	0.069	0.767
	First aid measures	0.89	**0.848**	0.023	0.184	0.753
	Storage conditions	0.84	**0.596**	0.511	0.135	0.635
	Expiry/production	0.79	**0.537**	0.223	0.246	0.399
Factor 2: Identity Measure	Barcode	0.81	0.132	**0.890**	0.046	0.811
	Batch number	0.90	0.075	**0.882**	0.024	0.783
	Manufacturing details	0.79	0.179	**0.731**	0.385	0.715
	Country of origin	0.83	0.269	**0.507**	0.340	0.516
Factor 3: Efficacy Measure	Product labelled with biocidal effect, e.g., antiseptic/disinfectant	0.80	0.201	0.077	**0.855**	0.778
	Product labelled with alcohol content 60%	0.86	0.030	0.113	**0.835**	0.711
	Correct spelling of active ingredient name (scientific name/brand name	0.79	0.362	0.159	**0.569**	0.480

PCFA employing MLA with oblimin rotation confirmed the three-factor model. The KMO value was found to be 0.812, and Bartlett’s test of sphericity had validity, i.e., *p*-value < 0.01. The non-salient factor loading distribution curve was found to be normal as a mean value of 0.1. The null-model χ2 was found to be 8237.23, while the implied model χ2 was 924.59. The PCFA for NFI was 0.970, CFI 0.978, and TLI 0.967. All values were in excess of 0.95, with RMSEA values below 0.06 at 0.03; all of these values indicated a good model fit.

### Reliability analysis

For this research, operationalized internal consistency (Cronbach’s alpha), item-internal consistency (IIC) and intraclass correlation consistency (ICC) were used to assess the study instrument’s reliability. All factors had a Cronbach’s alpha value in excess of 0.70; for the full scale, the value was 0.867.

In terms of item-internal consistency (IIC), the instrument demonstrated that every item met the IIC correlation standard ≥0.40. For intraclass correlation consistency (ICC), the scale displayed good overall ICC statistics of 0.867 (95% CI 0.856–0.877) with statistical significance (*p* < 0.001). All factors fell into an ICC range of 0.736 to 0.848. Factor 1 reliability was found to be 0.848 with a 95% confidence interval (0.835–0.861). For factor 2, the alpha value was 0.821, and the ICC was 0.804–0.836 (95% CI). For factor 3, the alpha value was 0.736, and the ICC was 0.710–0.760 (95% CI). Further details can be seen in Table 3. The scale’s test-retest reliability was assessed through correlation of the falsified hand sanitizer identification score of respondents at the two time points. The test-retest correlation coefficient was 0.770 (*p* value < 0.01).

**Table 3:** Reliability assessment criteria of the study scale (*n* = 1280)

Subscale	No. of items	Mean ± SD	Cronbach’s α	IIC	ICC (95% CI)
Factor 1	5	20.7 ± 4	0.848	0.66–0.87	0.848 (0.835–0.861)
Factor 2	4	9.4 ± 3.7	0.821	0.52–0.90	0.821 (0.804–0.836)
Factor 3	3	11.6 ± 3.1	0.736	0.78–0.83	0.736 (0.710–0.760)
Total	12	41.7 ± 8.6	0.867	–	0.867 (0.856–0.877)

Abbreviations: *SD* standard deviation, *IIC* item-internal consistency, *ICC* intraclass correlation (consistency ICC from 2-way mixed model)

### Known group validity

As shown in Table 4. The known group validity was assessed by one-way ANOVA. A statistically significant relationship between education levels and falsified hand sanitizer identification scores was reported. Participants with a post-graduate education were more likely to identify the falsified hand sanitizer compared to those with a high school education. (*p* < 0.001).

**Table 4:** Fake hand sanitizer identification scores by education level

Education level	Fake hand sanitizer identification scores			
	Mean	± SD	95% confidence interval	
			Lower limit	Upper limit
High school	41.37	± 8.7	40.78	41.96
Bachelor’s degree	41.60	± 7.5	40.86	42.34
Postgraduate	51.0	± 12.5	46.47	55.53

## Discussion

From the time the WHO announced that a global pandemic of COVID-19 had begun, falsified hand sanitizer began to appear more regularly on the market. These products pose a possible danger to public health, especially as they may have undeclared methanol among their ingredients. The current study developed and validated a novel tool to document falsified hand sanitizer in the UAE community (Supplementary material 1). The tool addressed multiple domains that act as determinants of falsified hand sanitizer.

When the regulatory authorities assess hand sanitizer products to ensure they are safe and compliant with health and safety regulations, they evaluate two elements, the label and the chemical composition of the product. To assess chemical composition, evaluation is undertaken in a laboratory to determine whether the levels of ingredients are at or below the maximum permitted limit, the pH of the product, the absence of any banned substances, and how efficacious the product may be.

In terms of assessing the label, it is compulsory for manufacturers to list the following information in the labelling: product name, brand, size, weight, indication, instructions for use, warnings, ingredients, storage conditions, production/expiry date, country of origin, manufacturer’s details, barcode and batch number. All of this information must be clearly noted on the hand sanitizer label If any of this information is lacking, then the product violates health and safety regulations and will be immediately removed from the market.

For this research, the instrument designed focused on the label requirements. The conceptual model comprised three dimensions: the safety of the product, its identity, and its efficacy. Respondents were asked to use a five-point Likert scale to state how often they checked the label information on hand sanitizers and ensured that it was legitimate.

The instrument used for this research underwent a number of stages for the assessment of validity. First, the items of the instrument were created by a specialist multidisciplinary committee to ensure that it was a suitable means of measuring. Following this, evaluation of a content validity index occurred, resulting in an acceptable content validity index value of 0.83. The instrument’s length and the time needed to complete it were regarded as a strong point that was responsible for a high response rate and high levels of respondent accuracy.

One aim of factor analysis was to assess whether it would be possible to harvest the underlying dimensions that were supportive of the conceptual model. Three distinct factors emerged from the analysis, as the model conceptualized. The factor analysis revealed three factors with eigenvalues in excess of 1, accounting for 67.8% of the total variance. The Kaiser-Meyer-Olkin (IMO) value was 0.788 with Bartlett’s test of sphericity achieving statistical significance (*p* < 0.001), supporting the factorability of the correlation matrix. Factor 1 evaluated the ability of the respondents to recognize the safety factors of the hand sanitizers, including usage instructions, warnings and first-aid measures. Factor 2 evaluated the ability of respondents to identify product identities for the hand sanitizer products, such as country of origin, details of manufacturer, batch number, and barcode. Factor 3 evaluated the ability of respondents to identify the efficacy of hand sanitizer. Following this, the 3-factor solution was confirmed by PCFA analysis. The 3-factor model solution had associations with good fit values, showing that the measurement purification for the scale was robust.

The reliability of the study instrument was proven. Overall, it satisfied the statistical criteria for reliability well. Additionally, there was a high percentage of fully completed questionnaires, which indicated that respondents had addressed the questions with care. The Cronbach’s alpha was good overall (0.867). For each domain, further analysis was undertaken regarding individual internal consistency, and relatively good alpha values for the three factors were found (0.736 to 0.848). There was satisfactory temporal stability in the instrument, demonstrated by the outcome of test-retest checks.

It was also shown that the instrument was capable of discriminating among groups. As predicted, scaled scores were significantly different depending on level of education. Respondents with higher education levels had significantly higher scores, demonstrating a greater ability to identify fake hand sanitizer.

If the appropriate authorities have the tools to recognize the issue, they will be able to implement programmes of education and awareness in relation to the hazards of falsified hand sanitizer and how it can be identified, which could result in a change in public attitude.

## Conclusions

This research developed and validated a new scale for the measurement of falsified hand sanitizer. A number of different methods were used to validate the tool, incorporating a number of domains that could be a useful means of self-reporting falsified hand sanitizer within the UAE. The survey is brief and simple and can be used to study attitudes in the general population. It may be of assistance to the appropriate regulators to be able to identify and rectify any issues that may prevent the public from being able to identify fake hand sanitizer. This could promote greater collaboration between the health regulators and the public, improve customer satisfaction and encourage the public to participate more with regard to this issue.

## Supplementary information

**Additional file 1: Supplementary material 1.** Validated Scale for the identification of falsified hand sanitizer (Questionnaire).

## Data Availability

The datasets generated during and/or analysed during the current study are available from the corresponding author on reasonable request.

## Abbreviations

AU: Ajman University
UAE: United Arab Emirates
IIC: Item internal consistency and intraclass correlation coefficients
ICCs: Intraclass correlation coefficients
CVR: Content validity ratio
CVI: Content validity Index
EFA: Exploratory factor analysis
PCA: Principal component analysis
KMO: Kaiser-Mayer-Olkin
PCFA: Partial confirmatory factor analysis
TLI: Tucker-Lewis index
NFI: Normed fit index
CFI: Comparative fit index
RMSEA: Root mean square error of approximation
