# Supplementary material for: Scale validation for the identification of falsified hand sanitizer: public and regulatory authorities perspectives from United Arab Emirates
Source: BMC Public Health. 2020 Oct 22;20:1595. doi: 10.1186/s12889-020-09707-0 (PMC7579848; doi:10.1186/s12889-020-09707-0)
Supplement: Supplementary file 1 — Additional file 1: Supplementary material 1. Validated Scale for the identification of falsified hand sanitizer (Questionnaire). [file 12889_2020_9707_MOESM1_ESM.docx]

**Scale Validation for the identification of falsified hand sanitizer: Public and Regulatory Authorities Perspectives from United Arab Emirates**

1. **Demographic and bassline characteristics**

| Variable | Groups |
| --- | --- |
| Sex | - Male |
|  | - Female |
| Age | - 18-24 |
|  | - 25-34 |
|  | - 35-44 |
|  | - 45-54 |
|  | - ≥ 55 |
| Nationality | - UAE |
|  | - Asia |
|  | - Africa |
|  | - America |
|  | - Middle East |
| Education | - High school |
|  | - Bachelor`s degree |
|  | - Post graduate |
| Employment status | - Employed |
|  | - Unemployed |

1. **Falsified hand sanitizer identification scale**

| Items | Response |
| --- | --- |
| *How frequently* you check the following information on hand sanitizer label when you buying hand sanitizer: | Never Rarely Sometimes often Always |
| Safety Measures |  |
| 1. Instruction of use, indication are clearly mentioned in the label such as:   *Dispense a required amount of hand sanitizer in you palm then briskly rub hands together until dry* | Never Rarely Sometimes often Always |
| 1. Warning and cautions are clearly indicated in the product label   *For external use only. Keep out of reach of children; avoid direct contact with eyes and mucous membranes, flammable*. | Never Rarely Sometimes often Always |
| 1. The first aid measure are clearly indicated on the label such as:   *In case of eye contact or skin irritation pleases consult a physician. If swallowed contact a poisons center* | Never Rarely Sometimes often Always |
| 1. The storage conditions are clearly indicated on the label such as: *Store at room temperature, keep away from fire or flam* | Never Rarely Sometimes often Always |
| 1. Expiry/production date are clearly indicated on the label |  |
| Identity Measures |  |
| 1. Barcode is indelibly impressed or imprinted on the product label | Never Rarely Sometimes often Always |
| 1. Batch number is indelibly impressed or imprinted on the product label | Never Rarely Sometimes often Always |
| 1. The manufacturer's name and logo Legible and correct | Never Rarely Sometimes often Always |
| 1. Country of origin is clearly indicated on the product label | Never Rarely Sometimes often Always |
| Efficacy Measures |  |
| 1. The product labeled with biocidal effect e.g. antiseptic/disinfectant | Never Rarely Sometimes often Always |
| 1. Product labeled with Alcohol content 60% | Never Rarely Sometimes often Always |
| 1. The active ingredient name spelt Correctly (scientific name/brand name) | Never Rarely Sometimes often Always |
